# Connecting G protein-coupled estrogen receptor biomolecular mechanisms with the pathophysiology of preeclampsia: a review

**DOI:** 10.1186/s12958-023-01112-7

**Published:** 2023-07-01

**Authors:** Allan Kardec Nogueira Alencar, Kenneth F. Swan, Gabriella Pridjian, Sarah H. Lindsey, Carolyn L. Bayer

**Affiliations:** 1 Department of Biomedical Engineering, Tulane University, 500 Lindy Boggs Center, New Orleans, LA 70118 USA; 2 Department of Obstetrics & Gynecology, Tulane University, New Orleans, LA 70112 USA; 3 Department of Pharmacology, Tulane University, New Orleans, LA 70112 USA

**Keywords:** Pregnancy, Preeclampsia, Estrogen, GPER, Extravillous trophoblast, Spiral arteries, Hypoxia, Angiogenesis, Uteroplacental circulation

## Abstract

**Background:**

Throughout the course of pregnancy, small maternal spiral arteries that are in contact with fetal tissue undergo structural remodeling, lose smooth muscle cells, and become less responsive to vasoconstrictors. Additionally, placental extravillous trophoblasts invade the maternal decidua to establish an interaction between the fetal placental villi with the maternal blood supply. When successful, this process enables the transport of oxygen, nutrients, and signaling molecules but an insufficiency leads to placental ischemia. In response, the placenta releases vasoactive factors that enter the maternal circulation and promote maternal cardiorenal dysfunction, a hallmark of preeclampsia (PE), the leading cause of maternal and fetal death. An underexplored mechanism in the development of PE is the impact of membrane-initiated estrogen signaling via the G protein-coupled estrogen receptor (GPER). Recent evidence indicates that GPER activation is associated with normal trophoblast invasion, placental angiogenesis/hypoxia, and regulation of uteroplacental vasodilation, and these mechanisms could explain part of the estrogen-induced control of uterine remodeling and placental development in pregnancy.

**Conclusion:**

Although the relevance of GPER in PE remains speculative, this review provides a summary of our current understanding on how GPER stimulation regulates some of the features of normal pregnancy and a potential link between its signaling network and uteroplacental dysfunction in PE. Synthesis of this information will facilitate the development of innovative treatment options.

## Background

Preeclampsia (PE) is a pregnancy-specific syndrome that is estimated to affect approximately 4–5% of pregnancies worldwide [1–3]. In developed countries, it is responsible for about 16–18% of maternal deaths and about 40% of fetal and neonatal deaths [4]. Classically, when pregnant women are diagnosed with PE, they present with new-onset hypertension and proteinuria after 20 weeks of gestation [5], but the disease may still be identified in the absence of renal dysfunction [6–8]. PE is a heterogeneous disease since its epidemiology and clinical presentation vary between early-onset PE, developing before 34 weeks of gestation, and late-onset PE, occurring after 34 weeks of gestation [9, 10]. This heterogeneity defines the two-stage model of PE [8] which is discussed later in this review.

Recent evidence reveals that PE induces short-term health consequences for both mother and child, with increased risk of cardiorenal disturbances in later life [11–13]. Therefore, targeted therapies with short- and long-term benefits are desperately needed, as delivery of the fetus and placenta remains the only definitive treatment [14]. Estrogens are sex hormones that act as crucial regulators of the female reproductive system, and their role in the maintenance of uteroplacental homeostasis has been documented in numerous preclinical and clinical studies [15–19]. Estrogen action is believed to be mediated by three estrogen receptors (ER): Estrogen receptor α (ERα), β (ERβ), and G protein-coupled estrogen receptor (GPER). To evaluate the impact of GPER on estrogen-induced regulation of pregnancy, it is essential to establish GPER’s autonomous function from the ER homologues in various aspects of pregnancy. In this work, we briefly revisit the key physiological features of pregnancy and pathophysiological mechanisms of PE. Since the pharmacological profile of GPER is currently under investigation in this field, we then discuss the current understanding of the biomolecular contributions of this metabotropic receptor towards normal placentation and pathogenesis of PE to shed new light on the potential benefits of selectively targeting GPER for the treatment of this obstetrical disease. In this narrative review, of all literature published through December 2022 was conducted using numerous primary topic headings combined with appropriate terms for each section of the article [e.g., pregnancy, preeclampsia, uteroplacental interface, estrogen, GPR30 or GPER, placentation, extravillous trophoblast, migration, invasion, endothelial dysfunction, oxidative stress, inflammation, hypoxia, angiogenesis]. Relevant full text articles published in English language were included in this manuscript.

## Biomolecular aspects of placentation and preeclampsia

### Physiological placentation

Normal early human placental development involves envelopment of the embryo inside the endometrial lining around day 10 post-conception. Under hypoxia and hypoglycemia, nutrition of the blastocyst is provided by secretions from the endometrial glands until the placental circulation is completely established [20]. At 8–10 weeks of gestation, placental extravillous trophoblasts (EVTs) undergo a phenotypic transformation into invasive cells [21]. This phenomenon occurs partially through an epithelial-to-mesenchymal transition, where epithelial-like adhesion molecules are replaced by vascular-like adhesion molecules [21]. Following this step, EVTs invade the decidualized endometrium to reach the inner third of the myometrium [22] and replace smooth muscle cells and elastin in the arteries [23]. Subsequently, EVTs invade and accumulate in the lumen of the spiral arteries to form ‘arterial trophoblast plugs’ [24]. This process occurs through the decidua and is fundamental for the development of the uteroplacental circulation, and usually occurs by 18 weeks of gestation [25–27]. Importantly, throughout the course of pregnancy, the small maternal spiral arteries dilate to become compatible with the increasing blood demands of the fetoplacental structure [28].

### Two-stage model of preeclampsia: abnormal placentation

In placentas that develop PE, EVTs fail to transform from the proliferative epithelial to the invasive phenotype, which is the main cause of incomplete remodeling of the spiral arteries [21]. Dysfunction in spiral artery remodeling leads to narrowing of uterine vessels and compromises placental blood flow [29, 30]. EVT abnormalities result in shallow placentation and insufficient remodeling of the spiral arteries, which triggers subsequent ischemia of this organ in the first stage of PE [21].

Secondary to shallow EVTs invasion, the ischemic and structurally-damaged placenta releases factors into the systemic circulation in an attempt to increase blood flow and oxygen delivery to the fetus. However, these factors also increase oxidative stress in syncytiotrophoblasts (STBs), a continuous, specialized layer of epithelial cells covering the chorionic villi [31]. Stressed STBs release proinflammatory cytokines and antiangiogenic factors into the systemic maternal circulation, and injuring molecules that damage the mother’s vasculature (mainly the endothelium) [32–34]. This second stage of PE is characterized by a substantial injury of the maternal vascular endothelium and stimulation of an inflammatory response, culminating in clinical symptoms [35, 36].

### Pro-oxidant and inflammatory components of preeclampsia

The uteroplacental interface undergoes a pro-oxidant stage in the first weeks of normal pregnancy, as the increase in the metabolic rate ensuring adequate fetal development comes together with oxidative stress in the placental tissues. This period of gestation is also characterized by the high expression and activity of antioxidant enzymes to maintain oxidative balance [37].

The neutralization of reactive oxygen species (ROS) by antioxidant enzymes is disturbed and oxidative stress is significantly exacerbated in PE [38]. It is suggested that impaired perfusion due to aberrant remodeling of uterine arteries induces placental oxidative stress [39]. For example, impaired perfusion leads to repeated events of hypoxia/reoxygenation, which in turn triggers oxidative stress in the placenta, and the increasing amount of ROS might damage the DNA and induce low-density lipoprotein oxidation, with subsequent lesion and/or cell death [39]. Importantly, oxidative stress in PE stimulates the synthesis of proinflammatory cytokines such as tumor necrosis factor alpha (TNF-α) and interleukin 6 (IL-6), with a simultaneous reduction in anti-inflammatory cytokine production, such as interleukin 10 (IL-10) [40]. An immunologic imbalance is observed in the preeclamptic decidua, where the secretion of TNF-α and IL-6, and a decrease of immune cells that normally facilitate trophoblast migration [e.g., macrophages, natural killer (NK) cells, T cells, and regulatory T cells (Tregs)] occur [39, 40]. Additionally, this imbalance activates macrophages and neutrophils, inflammatory cells that convert oxygen into superoxide radical anions (O_2_^•−^), ROS molecules that damage the placenta [39].

### Hypoxia and angiogenic disturbances in preeclampsia

It is generally accepted that placentation and embryonic development under hypoxia are not pathological events, as low oxygen (O_2_) levels in early gestation expose the blastocyst to severe hypoxia in the uterus at day 6 post-conception [41]. This microenvironmental hypoxia is maintained for up to 10 weeks of pregnancy and occurs when the spiral arteries become plugged to avoid the blood flowing from the maternal circulation into the intervillous space [41]. By the end of first trimester, this plug is dissolved, and the maternal arteries fully enter the intervillous space when then the O_2_ level raises to a “physiological” state [41]. This process of hypoxic-ischemic/reoxygenation is essential for fetal and placental development [41]. However, it has been recently reported that the hypoxic-ischemic/reoxygenation state leads to the formation of misfolded and aggregated proteins, resulting in excessive endoplasmic reticulum stress and an overactivated unfolded protein response. These conditions create a state of proteotoxic stress that surpasses the proteostatic capacity of primary human placental trophoblasts, leading to placental insufficiency and the onset of preeclampsia-like symptoms [42].

Hypoxia-inducible factors (HIFs) are crucial transcription factors that regulate responses to hypoxia and are important molecules in both physiological and pathophysiological processes [43]. HIFs consist of the HIF-α subunit (HIF-1α or HIF-2α) and HIF-1β, and only the α subunit is regulated by O_2_ levels [43]. In an O_2_-depleted state, the α-subunit is translocated to the nucleus and activates the expression of target genes, thus mediating key cellular effects in response to hypoxia, such as angiogenesis, cell migration/invasion, and immune cell function [43]. As pregnancy progresses, HIF-1α protein levels gradually decrease, and are almost undetectable by week 12 [44]. However, placental hypoxia eventually persists beyond the first trimester in PE, as the expression of HIFs is elevated throughout gestation [45].

The pivotal role of hypoxia in PE has been reviewed by Hu et al. [45], where they discussed seminal studies with humans and animals that experienced hypobaric and/or normobaric hypoxia. As summarized by the authors, persistent hypoxia during pregnancy increases placental HIF expression, boosts HIF synthesis in trophoblast cells, and inhibits the invasive potential of EVTs, with impaired spiral artery remodeling observed due to prolonged expression of trophoblast-specific HIF-1α [45]. Further analysis showed that pregnant mice overexpressing HIF-1α exhibit PE phenotype [46].

The production of soluble Fms-like tyrosine kinase-1 (sFlt-1) by trophoblasts is triggered during persistent hypoxia in PE as a transcriptional response induced by high levels of HIF-1α and HIF-2α [47, 48]. sFlt-1 has anti-angiogenic properties and is significantly increased in blood samples from PE patients [49], contributing to disease pathogenesis by inducing endothelial dysfunction, disrupting angiogenesis, and impairing trophoblast invasion [49]. sFlt-1 binds to vascular endothelial growth factor (VEGF) and placental growth factor (PlGF) with high affinity and inhibit their activity on vascular endothelial cells, which might impair the vascular growth of spiral arteries [50]. Transgenic animal models show that an increase in circulating levels of sFlt-1 and decrease in bioavailability of PlGF results in signs of PE (e.g., hypertension and proteinuria), demonstrating the causal role of this pathway in disease pathophysiology [51].

### Vascular dysfunction in preeclampsia

An adaptive switch in the uteroplacental vasculature from pro-angiogenic stimulation of new vessel growth to vasodilation occurs as gestation progresses [52]. Specifically, from mid-gestation to parturition, necessary blood supply to the fetus is highly dependent on endothelium-induced vasodilation in uteroplacental vessels [52]. As elegantly reviewed by Opichka et al., the imbalance between constriction and relaxation, and hemodynamic modifications that alter body fluid homeostasis are features of PE [53]. Endothelial dysfunction, specifically in the form of barrier disruption and impaired vasodilatory capacity, is prevalent in PE and is implicated in many stages of the disease [53]. Late-stage PE is characterized by vascular defects thought to be targeted to the endothelium of some vascular beds, since the incubation of myometrial arteries with preeclamptic plasma impairs vasorelaxation in endothelium-denuded but not in intact vessels [54]. An in vivo study found that flow-mediated dilation is reduced in women with previous PE compared with normal pregnancies [55]. Authors highlight that flow-mediated dilation is an endothelium-dependent phenomenon, what indicates that these findings are endothelial-specific [55].

Generally, the decreased synthesis of relaxing substances such as nitric oxide and prostacyclin and increased vasoconstriction induced by angiotensin II, endothelin-1 and vasopressin are considered pathogenic mechanisms of PE [56, 57]. It has been demonstrated that vascular resistance regulates the systemic circulation and has significant effects within specific vascular beds [54, 55, 58–60]. For example, uteroplacental arteries from preeclamptic women produce less endothelial-derived vasodilatory molecules than that of women with uncomplicated pregnancies, and this may be related to oxidative stress [54, 55, 60]. Therefore, the exchange between placenta and fetus is negatively affected by the uteroplacental resistance, whereas systemic resistance contributes to an array of multiorgan dysfunction in PE, such as glomerular endotheliosis, liver failure, and central nervous system damage [53].

## Connecting GPER effects with the pathobiology of preeclampsia

It is noteworthy that diethylstilbesterol, a potent ERα and ERβ agonist, has a low binding affinity for GPER and is associated with various adverse side effects, including PE [61, 62]. Similarly, estriol, which is produced in large quantities by the placenta, also has low affinity for GPER and even acts as a GPER antagonist at micromolar concentrations [63]. This suggests that the lower affinity of these estrogenic hormones for GPER may be advantageous in the context of PE, a condition where GPER is believed to play a role. The fact that these estrogenic hormones do not strongly activate GPER signaling may help prevent excessive GPER activity that may contribute to the development of PE. Therefore, it is possible that GPER-selective compounds may have therapeutic potential for the treatment of PE.

An extensive body of literature has characterized GPER as predominantly responsible for the rapid actions of estrogen [64–68], its effects on gene expression have also been described [69–72]. When estrogen stimulates GPER, the transient activation of heterotrimeric G proteins intermediates several downstream signaling events [62, 73] that are propagated to the nucleus to modulate transcription factors [74]. The ultimate cellular response to estrogen results from a complex interplay between transcriptional and non-transcriptional phenomenon [75].

GPER is expressed in several cell types in humans [76–81] and rodents [82–89]. Examples of organs/tissues in which GPER is expressed are the brain, lungs, prostate, liver, ovaries, placenta, pancreas, adipose tissue, vasculature, skeletal muscles, heart, kidneys, and immune cells [90–92]. A diverse number of disorders are related to the aberrant expression and function of GPER [67, 93], and advances in our understanding of the pathogenic roles of GPER in PE offer opportunities for targeting this process in the development of early disease interventions. For example, estrogen receptor knockout models have played a crucial role in identifying and evaluating the biological significance of GPER. To strengthen the claim that GPER is vital in PE, it is imperative to examine the pathological changes that occur in estrogen receptor-deficient mice, such as hypertension, atherosclerosis, and renal dysfunction, as these are defining features of PE.

The primary focus of research on GPER’s vascular effects has been on its impact on vascular reactivity and blood pressure in the short term. When GPER is selectively activated with the G-1 agonist in isolated vessels, it causes vasodilation in carotid vessels of mice but not in those of GPER knockout mice [94]. Activation of GPER results in both acute and chronic reduction in blood pressure in ovariectomized mRen2.Lewis rats [94, 95], while the absence of GPER due to genetic deletion leads to elevated blood pressure in female mice. Although estrogen does not decrease plasma cholesterol and lesion size in mice lacking ERα [96], it is still able to reduce advanced lesion characteristics. Interestingly, in intact and ovariectomized female GPER knockout mice, aortas exhibited an exacerbation of lesion size, implying that GPER may play a beneficial role in the context of atherosclerosis [97]. These findings suggest that while ERα is likely the main mediator of atherosclerotic protection, GPER may also contribute to protective mechanisms. It is also crucial to emphasize that estrogen offers protection against renal damage in mice. However, the absence of ERα or ERβ genes does not weaken this safeguard [98], indicating that GPER may serve as an alternative receptor that provides estrogen-induced protection during kidney disturbances. The genetic modifications mentioned above emphasize the potential significance of GPER as a therapeutic target for cardiorenal disorders, specifically in the context of PE. As such, the following sections of this review will examine the linkages between GPER signaling pathways and the pathophysiology of PE. This will be achieved by delving into relevant literature, identifying gaps in our understanding, and addressing points of controversy.

### The role of GPER in the pathophysiology of cancer and its correlation with preeclampsia

An analysis of the similarities and differences between the physiological state of pregnancy and the pathological state of cancer is significant as it may aid in identifying potential therapeutic targets to treat PE, with a particular focus on GPER. Numerous reviews have investigated the potential role of G-protein coupled receptors (GPCRs) in cancer [99–101]. These receptors are essential in regulating metabolism, energy, and tissue homeostasis, which are critical physiological responses that cancer cells exploit. Furthermore, GPCRs are often likened to a “chronic wound” in the context of cancer, given their involvement in cellular processes that facilitate inflammation, tissue remodeling, and angiogenesis [102, 103] similar to those observed during normal placentation [104]. Within this paradigm, GPER’s participation in estrogen-induced carcinogenesis is postulated based on the view that cancer is a chronic wound caused by imbalanced glandular epithelial homeostasis [105]. Consequently, GPER stimulates estrogen-induced carcinogenesis by triggering intracellular signaling pathways that allow for malignant cells utilize several molecular mechanisms found in trophoblastic cells, such as migration and invasion, angiogenesis, immune tolerance, proliferation, differentiation, apoptosis, and survival, to establish a supportive environment, avoid apoptosis, and elude the host immune response [105–108].

### GPER and extravillous trophoblast invasion

Although EVTs are highly invasive in the early stages of pregnancy, this phenotype progressively decreases to avoid excessive invasion of placental tissue in the uterus [109]. Importantly, Tong et al. reported that GPER is expressed in human EVTs at different stages of pregnancy (first trimester and term placentas) and modulates EVT function [109]. Additionally, placentas collected at term from PE women present a dramatic reduction in GPER expression, which may be a causative factor in disease pathogenesis [109]. Furthermore, it was demonstrated that GPER levels in EVTs could be upregulated by estrogen treatment, which implies that the reduced expression of GPER is probably attributed to impaired estrogen synthesis in PE placentas [109].

The migratory potential of EVTs is triggered by matrix metalloproteinases (MMPs), cathepsins, and urokinase plasminogen activator, which are biomolecules that degrade the extracellular matrix of uterine tissue and facilitate EVT invasion [110, 111].

Tong et al. further elucidated the mechanisms underlying GPER-mediated EVTs invasion when they cultured and incubated an immortalized human trophoblast cell line (HTR8/SVneo) with G-1 and estrogen [109]. Activation of GPER with both G-1 and estrogen increased the expression of MMPs, specifically MMP-9, in HTR8/SVneo cells [109]. Intriguingly, co-incubation of HTR8/SVneo cells with G15, a selective antagonist of GPER, significantly inhibits the expression of MMP-9 [109]. Thus, the authors of this study proposed that MMP-9 is a downstream effector of GPER in EVTs invasion [109].

**Fig. 1:**
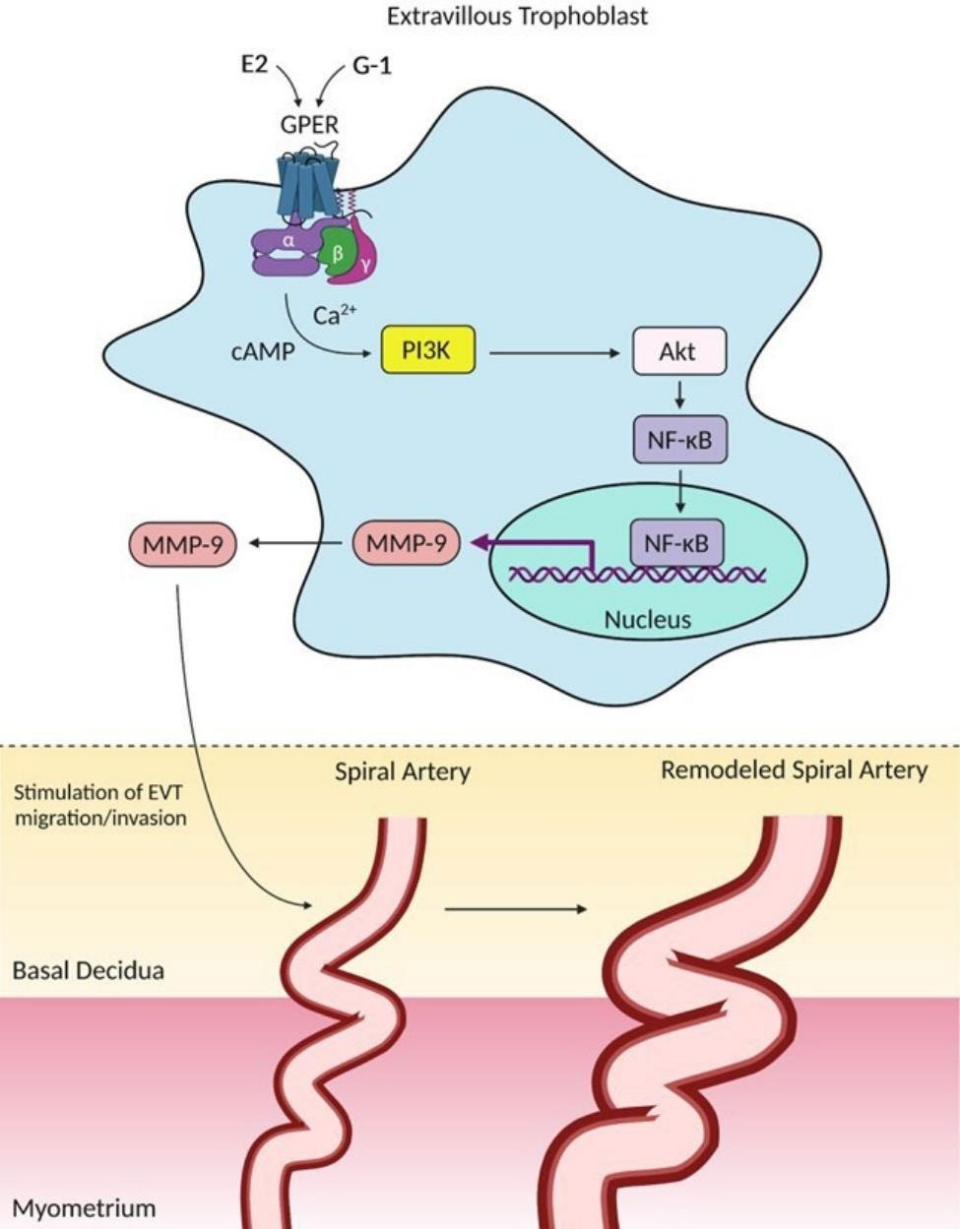
Overview of GPER signaling involved in the modulation of EVT migration/invasion through the PI3K/Akt-MMP-9 axis. Pharmacological modulation of GPER by E2 or its selective agonist G-1 stimulates distinct subunits of heterotrimeric G proteins. G_αq_ and G_αs_ are examples of subunits stimulated by GPER, which augment the intracellular levels of second messengers (Ca^2+^ and cAMP) to promote activation of PI3K/Akt enzymes. Once activated, PI3K/Akt cascade triggers NF-κB translocation to the nucleus, where it encodes the synthesis of MMP-9, a downstream effector of GPER-regulated EVT cell migration/invasion and subsequent spiral artery remodeling. Additional mechanisms involved in the GPER/PI3K/Akt/MMP-9 downstream signaling pathway are provided in this figure and have been published elsewhere [140, 141]. GPER, G protein-coupled estrogen receptor; EVT, extravillous trophoblast; PI3K, phosphoinositide 3-kinase; Akt, protein kinase B; MMP-9, matrix metalloproteinase 9; E2, estrogen; cAMP, cyclic adenosine monophosphate; NF-κB, nuclear factor-κB. This artwork was created using the BioRender software

Neoplastic cells invade tissues and metastasize through the activity of MMPs that are upregulated by the phosphoinositide 3-kinase (PI3K)/protein kinase B (Akt) pathway [112, 113]. Remarkably, both G-1 and estrogen significantly augment the phosphorylation of PI3K and Akt proteins in HTR8/SVneo cells, whereas activation of the PI3K/Akt pathway was attenuated by G15 [109]. As discussed by the authors, the response of PI3K/Akt to GPER modulation is consistent with increased MMP-9 expression, which suggests that PI3K/Akt could be coupled with MMP-9 expression in trophoblasts to mediate GPER-regulated cell invasion [109] (Fig. 1).

A recent study investigated the additional mechanisms by which GPER influences EVTs invasion [114]. In this original research, scientists applied an RNA sequencing technique to HTR8/SVneo human trophoblast cells to investigate the relationship between GPER and angiopoietin-like 4 [114]. Angiopoietin-like 4 is a protein encoded by ANGPTL4 gene [115]. The key finding of this study was the identification of ANGPTL4 as a target gene for GPER in EVTs cells [114].

The activation of Hippo tumor-suppressor pathway (Hippo pathway) stimulates mammalian serine/threonine kinases STE20-like 1 and 2 (MST1/2), which, in turn, phosphorylate the downstream large tumor suppressor 1 and 2 kinases (LATS1/2) [114]. Thus, phosphorylated LATS1/2 subsequently phosphorylates Yes-associated protein (YAP), the major downstream effector of the Hippo pathway [114]. This intracellular signaling results in cytoplasmic retention of YAP and its proteolytic degradation [114]. However, when the Hippo pathway is inhibited, YAP is dephosphorylated, which prevents its export from the nucleus and promotes its transcriptional activity by interaction with TEA domain protein family of transcription factors [114]. Within the Hippo pathway, phosphorylation dependent on LATS1/2 is thought to be the most important event in the regulation of YAP signaling activity [116]. This can be explained by the fact that preclinical knockout of LATS1/2 abolishes most YAP phosphorylation in response to many known upstream regulatory signals [116].

**Fig. 2:**
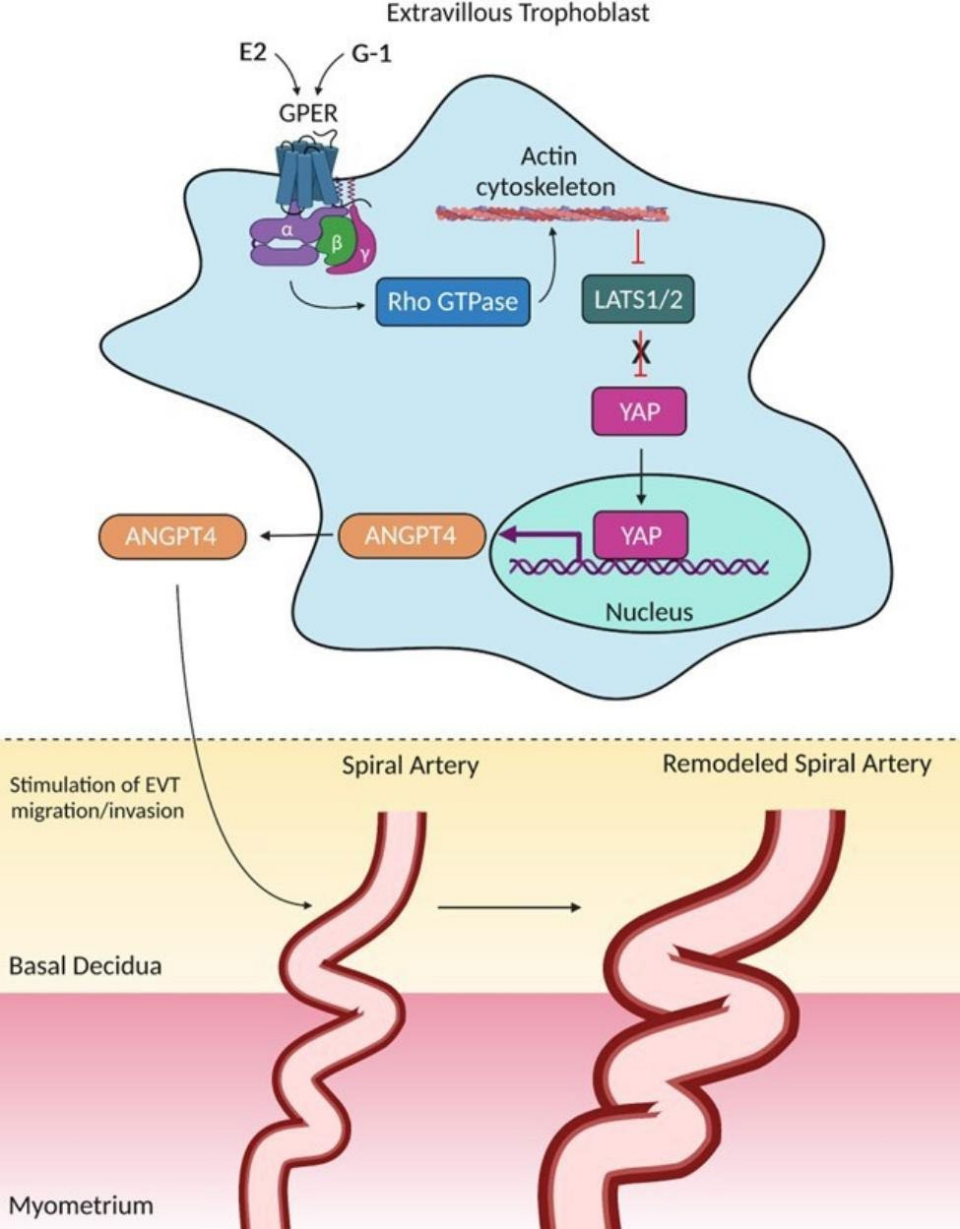
Molecular mechanisms by which GPER stimulates EVT migration/invasion through the Hippo pathway. When GPER is activated by E2 or G-1, its G_αq_ subunit stimulates Rho GTPase, which in turn causes actin cytoskeleton organization, a crucial regulator of the Hippo pathway. Actin cytoskeleton inhibits LATS1/2 activity, thus increasing the translocation of YAP protein to the nucleus. Disruption of actin cytoskeleton or inhibition of Rho GTPase facilitate the phosphorylation/activation of LATS1/2 and subsequent inhibition of YAP nuclear translocation and activity. This results in cytoplasmic retention of YAP and its proteolytic degradation. However, the inhibition of LATS1/2 by the actin cytoskeleton is a crucial mechanism responsible by YAP transcriptional activity in the nucleus, where this protein encodes the synthesis of ANGPTL4. When ANGPTL4 is produced, it modulates the EVT cell migration/invasion and subsequent spiral artery remodeling. Additional mechanisms involved in the GPER-induced ANGPTL4 synthesis by the Hippo pathway are provided in this figure and have been published elsewhere [142]. GPER, G protein-coupled estrogen receptor; EVT, extravillous trophoblast; Hippo pathway, Hippo tumor-suppressor pathway; LATS1/2, large tumor suppressor 1 and 2 kinases; YAP, Yes-associated protein; ANGPTL4, angiopoietin-like 4. This artwork was created using the BioRender software

YAP is expressed in human EVTs cells and plays a pivotal role in the maintenance of cell proliferation and stemness [117]. Interestingly, Cheng et al. showed that YAP expression and activity were reduced in PE EVTs compared to control cells [114]. Moreover, the transwell invasion assay showed that GPER and YAP are required for G-1- or estrogen-induced EVTs invasion [114]. Accordingly, these data indicate that downregulation of GPER and YAP contributes to PE by impairing trophoblast cell invasion [114]. In this study, researchers have provided further evidence that angiopoietin-like 4 mediates GPER-stimulated trophoblast cell invasion and that downregulation of this protein triggers a dysfunctional invasion effect in these cells [114] (Fig. 2).

### GPER and angiogenesis/hypoxia in preeclampsia

The growth and development of the conceptus is aided by the endometrial glands, which secrete various substances such as glycogen, lipid droplets, and glycoproteins (such as glycodelin, and osteopontin). These substances provide essential nutrition, facilitate immune reactions and cell migration, while cytokines and growth factors [such as epidermal growth factor (EGF) and VEGF] promote the proliferation and angiogenesis required for placental development [118]. In this regard, activation of GPER has been shown to play a role in the activation of the EGF receptor (EGFR) in cancer cells, which provides insight into its potential role as a regulator of angiogenesis in placental development. GPER stimulates the downstream signaling pathway of EGFR through transactivation [64], which is achieved by an EGFR ligand-dependent pathway. The transactivation process involves an increase in MMP expression by GPER, leading to the release of membrane-anchored EGFR ligands. In this pathway, GPER activation leads to the dissociation of the G-βγ complex and subsequent activation of the Src-related tyrosine kinase family downstream, along with phosphorylation of the Shc adapter protein, which enhances MMP expression and activity in the cell membrane, which in turns leads to the release of heparin-binding epidermal growth factor [67, 119, 120]. Therefore, we can infer that MMPs are not simply are secreted by the cells. In this particular instance, their actions are influenced by the plasma membrane, specifically in the release of membrane-tethered EGF-like ligands. The release of these ligands subsequently activates EGFR and triggers both the mitogen-activated protein kinase (MAPK)/PI3K and Akt pathway in cancer cells, leading to increased proliferation and angiogenesis [67, 119, 120]. This information is intriguing as it suggests a possible role of GPER-induced EGFR transactivation in aiding placental development.

The biomolecular roles of GPER in hypoxia and angiogenesis during PE have not been dealt with in-depth yet, with only few studies presenting general data such as changes of GPER expression levels in HTR8/SVneo cells submitted to hypoxia-reoxygenation [121] and GPER role in modulating the imbalance between proliferation and apoptosis induced by hypoxia-reoxygenation in trophoblast cells [122]. Molecularly, research is also needed to determine possible effects of GPER in the regulation of expression and activity of key markers of hypoxia and angiogenesis in PE (e.g., HIF-1α and VEGF). Since normal placentation exhibits many features common to cancer [123], here we outline some important signaling pathways described for GPER in malignant cells that could be exploited in PE.

The relationship between GPER and HIF-1α seems to be cycle-regulated, as some studies show that GPER expression is increased by HIF-1α [124, 125], and that HIF-1α is up-regulated by GPER [126, 127]. Bioinformatic analysis has shown the presence of a hypoxia-responsive element located within the promoter region of GPER gene in tumor cells [124], and De Francesco et al. found a functional cooperation between HIF-1α and GPER in breast cancer cells associated fibroblasts [127]. They have shown that a low O_2_ tension upregulates HIF-1α which, in turn, increases the expression of GPER, and that these both molecules are recruited to the hypoxia-responsive element site located within the VEGF promoter region and cooperatively act as a functional complex for the transcription of VEGF and induction of tumor angiogenesis [127]. De Francesco et al. further highlighted that their results may also disclose an estrogen-independent action elicited by GPER [127].

As addressed earlier in the present work, HIF-1α levels are increased throughout pregnancies complicated by PE. Therefore, intriguing questions arise: (1) If HIF-1α stimulates the transcription of GPER independently of estrogen agonism in malignant cells, why is the GPER expression reduced in hypoxic placentas? (2) Shouldn’t the relationship between HIF-1α and GPER be cycle-regulated in the PE context as well? Further identifications of context-specific HIF-1α and GPER interaction pattern could be crucial for responding to these questions and developing targeted therapies for PE.

### Regulation of systemic vs. uteroplacental vascular tone by GPER

Accumulating findings have been well described and reviewed in the literature, concerning the roles triggered by GPER in maintaining the homeostasis of the cardiovascular system [95, 128–132]. Since the mesenteric vascular bed significantly contributes to the total peripheral resistance [133] and both structural and functional alterations in mesenteric vessels are involved with the pathogenesis of systemic hypertension [134–136], it would be of great importance to differentiate the GPER profile between mesenteric and uterine vasculature from nonpregnant, normal pregnant and preeclamptic subjects. In this regard, Mata et al. have published the first study that investigated GPER expression and its vasodilator activity in a blood vessel-specific pattern during pregnancy in rats [137]. They found that GPER expression does not change in mesenteric vasculature when compared between pregnant and nonpregnant rats [137]. Furthermore, they showed that G-1 promoted vasodilation in a concentration-dependent manner, but with no significant difference in the mesenteric vasculature of pregnant vs. virgin rats [137]. More recently, it was found that GPER is greater expressed in uterine radial arteries from pregnant rats than in nonpregnant [138]. The authors of this study also showed that G-1 promotes relaxation of isolated radial uterine arteries, and that its vasodilatory effect was more pronounced in vessels from pregnant than that in nonpregnant animals [138], what establishes a role of GPER in the regulation of rat uteroplacental vascular tone. In order to better support their conclusions, the same research group have shown that GPER-mediated vasodilation in rat uterine arteries is vascular-bed specific and correlated with gestational age [139]. In this study, G-1 elicited vasodilation in mesenteric arteries with a similar potency compared between nonpregnant and pregnant rats [139], contrary to the findings of G-1 in the uterine vasculature where its vasodilatory profile was significantly higher in vessels from pregnant (at different gestation periods) vs. nonpregnant animals [139]. The authors attributed this vascular-bed specific effect of G-1 to the differences in GPER expression amongst mesenteric and uterine vasculature since they found no changes in GPER levels in mesenteric arteries from nonpregnant vs. pregnant rats, but they did find that GPER is greater expressed in uterine arteries from pregnant than in nonpregnant rats, suggesting again that pregnancy-induced modulation of GPER is specific to uterine arteries [139].

GPER vasodilation in rat uterine arteries has been found to be endothelium-dependent and mediated by the nitric oxide-cyclic guanosine monophosphate (cGMP) axis [138] considering that G-1 effect was abolished after removal of the endothelium and inhibition of nitric oxide production with a further significant reduction of its vasodilatory efficacy shown after inhibition of cGMP synthesis [138]. Moreover, it has been recently described a possible smooth muscle-related mechanism involved in the uterine vascular responses to G-1 [139]. Interestingly, this original research showed that the blockage of L-type calcium channels caused a three-times reduction of the G-1-induced vasorelaxation in rat uterine arteries and inhibition of extracellular signal-regulated protein kinases 1 and 2 (ERK1/2) protein attenuated G-1 response by 24%, which is suggestive of a partial contribution of ERK1/2 pathway in the mechanism of action of GPER in uterine arteries [139]. Accordingly, these findings are supportive of a physiological role of GPER in the uterine circulation adaption to pregnancy.

## Conclusion

In this review, we have discussed the link between GPER activity and some of the key pathophysiological features of PE. It is evident that the roles of GPER in the regulation of uteroplacental cell functionality in normal pregnancy and in the preeclamptic environment are largely unknown. The successful characterization of GPER as a pharmacological target to treat PE requires significantly more research into what determines its potential of modulating biomarkers of oxidative stress, hypoxia, angiogenesis, inflammation and vascular dysfunction. Since most of the studies that are designed to clarify the mechanisms by which GPER affects uteroplacental biology are performed in vitro, it will be important to unravel its roles in different in vivo models of PE, as well as in normal pregnancy.

## Data Availability

Not applicable.

## Abbreviations

Akt: Protein kinase B
ANGPTL4: Angiopoietin-like 4
cAMP: Cyclic adenosine monophosphate
cGMP: Cyclic guanosine monophosphate
ERK1/2: Extracellular signal-regulated protein kinases 1 and 2
EGF: Epidermal growth factor
EGFR: Epidermal growth factor receptor
Erα: Estrogen receptor alpha
ERβ: Estrogen receptor beta
EVTs: Extravillous trophoblasts
GPER: G protein-coupled estrogen receptor
HIF-1α: Hypoxia-inducible factor 1 alpha
HIF-1β: Hypoxia-inducible factor 1 beta
HIF-2α: Hypoxia-inducible factor 2 alpha
HIFs: Hypoxia-inducible factors
Hippo pathway: Hippo tumor-suppressor pathway
HTR8/SVneo: Immortalized human trophoblast cell line
IL-10: Interleukin 10
IL-6: Interleukin 6
LATS1/2: Large tumor suppressor 1 and 2 kinases
MAPK: Mitogen-activated protein kinase
MMP-9: Matrix metalloproteinase 9
MMPs: Matrix metalloproteinases
MST1/2: Mammalian serine/threonine kinases STE20-like 1 and 2
NF-κB: Nuclear factor-κB
NK cells: Natural killer cells
O_2_: Oxygen
O_2_^•−^: Superoxide radical anions
PE: Preeclampsia
PI3K: Phosphoinositide 3-kinase
PlGF: Placental growth factor
ROS: Reactive oxygen species
sFlt-1: Soluble Fms-like tyrosine kinase-1
STBs: Syncytiotrophoblasts
TNF-α: Tumor necrosis factor alpha
Tregs cells: Regulatory T cells
VEGF: Vascular endothelial growth factor
YAP: Yes-associated protein
